# CT enterography for evaluation of disease activity in patients with ileocolonic Crohn's disease

**DOI:** 10.1186/s12876-022-02389-5

**Published:** 2022-06-30

**Authors:** Jinlu Tong, Qi Feng, Chenpeng Zhang, Xitao Xu, Zhihua Ran

**Affiliations:** 1 Division of Gastroenterology and Hepatology, NHC Key Laboratory of Digestive Diseases, Shanghai Inflammatory Bowel Disease Research Center, Shanghai Institute of Digestive Disease, Renji Hospital, Shanghai Jiao Tong University School of Medicine, Shanghai, China; 2 Department of Radiology, Renji Hospital, School of Medicine, Shanghai Jiao Tong University, Shanghai, China; 3 Department of Nuclear Medicine, Renji Hospital, School of Medicine, Shanghai Jiaotong University, Shanghai, China

**Keywords:** CT enterography, Disease activity, Crohn's disease, Ileocolonic

## Abstract

**Background:**

CT enterography (CTE) is used routinely for assessment of activity and severity in Crohn's disease (CD), but there are few CTE scoring systems. The aim of this study was to develop a quantitative CTE scoring system for ileocolonic Crohn's disease activity.

**Methods:**

Forty-nine CD patients with ileocolonic involvement were retrospectively included between March 2015 and May 2018. All patients underwent CTE and ileocolonoscopy. Mural hyperenhancement and mural thickening at CTE were scored quantitatively, while mural stratification, submucosal fat deposition, comb sign, perienteric fat hypertrophy and mesenteric fibrofatty proliferation were qualitative variables. A Tobit regression model was applied for assessing the association between Crohn's disease endoscopic index of severity (CDEIS) and CTE variables.

**Results:**

A total of 280 intestinal segments were evaluated. Independent predictors for CDEIS were mural thickness (p < 0.001), mural stratification (p < 0.001) and comb sign (p = 0.002). In order to quantify disease activity based on CTE findings in each segment, a simplified CT enterography index of activity (CTEIA) was derived from logistic regression analysis. The formula was as follows: CTEIA (segment) = 2.1 mural thickness(mm) + 9.7 mural stratification + 5.2 comb sign. There was a high and significant correlation coefficient between CDEIS and CTEIA (r = 0.779, p < 0.001) for per-segment analysis. The model for the detection of ulcerative lesions in the colon and terminal ileum achieved an area under the receiver-operating curve of 0.901 using a cut-off point of 6.25.

**Conclusions:**

CTEIA is a new qualitative tool for evaluation of ileocolonic Crohn’s disease, which need to be validated in further studies.

**Supplementary Information:**

The online version contains supplementary material available at 10.1186/s12876-022-02389-5.

## Background

Crohn’s disease (CD) is a chronic, progressive, inflammatory and disabling condition that can involve any segment in the gastrointestinal tract with symptoms evolving in a relapsing and remitting manner. The implementation of the “treat to target” strategy need a tight control of disease activity and adjusting therapy accordingly [1], also for monitoring following withdrawal of maintenance treatment [2]. Crohn's disease endoscopic index of severity (CDEIS) and simple endoscopic score for Crohn's disease (SES-CD) are considered as reference standards to assess mucosal healing in patients with Crohn's disease. Radiological examinations also play a major role in the diagnosis, management, and monitoring of Crohn’s disease and are complementary to endoscopic techniques, especially for small bowel lesions and intestinal stenosis. CT enterography (CTE) and magnetic resonance enterography (MRE) are cross-sectional imaging techniques that are optimized for imaging of the small bowel. MRE has become most accepted modality for the assessment of CD by considering non-ionizing radiation. Many studies have focused on developing MRE-based indices for quantification of active disease which will help in guidance of patients’ therapy, such as the Magnetic Resonance Index of Activity (MaRIA) [3], the Clermont score [4], the Crohn’s Disease magnetic resonance imaging index (CDMI) [5] and the magnetic resonance enterography global score (MEGS) [6]. However, limitations of MR enterography in comparison with CT include higher cost, less availability, more variable image quality, and lower spatial resolution [7, 8].

CT enterography is still widely used as first cross-sectional enterography exam [9], and there existed many semi-quantitative scoring systems for assessment of disease activity in patients with Crohn's disease [10]. Unfortunately, few quantitative CTE scoring system was developed for ileocolonic Crohn's disease activity.

## Patients and methods

This was a retrospective study performed at a single tertiary inflammatory bowel disease (IBD) referral center and was approved by the Ethics Committee of Renji Hospital (2020-031).

### Patients and examinations

All patients had established diagnosis of CD according to Lennard–Jones criteria. Using the hospital radiology information system, endoscopy database and patient database, we identified eligible patients with established CD who underwent CTE scans between March 2015 and May 2018, and each patient had performed CTE and ileocolonoscopy within 24 h. Clinical disease activity based on the calculation of the Harvey–Bradshaw index (HBI), laboratory tests, as well as concomitant therapy at the time of examination were collected based on patients’ record files.

Patient inclusion criteria were as follows: (1) clinical diagnosis of CD; (2) age should be 18 years and older; (3) contrast enhanced CTE examination conducted between March 2015 and May 2018; (4) ileocolonoscopy within 24 h of the date of CTE. Exclusion criteria were: (1) patients younger than 18 years; (2) pregnant women; (3) patients had low quality CTE images.

### Endoscopic data collection

The severity and extent of inflammatory lesions were evaluated using CDEIS [11]. For the CDEIS, the endoscopic parameters such as (i) presence or absence of deep and superficial ulcers, (ii) percentage of ulcerated or affected surface (evaluated on a 10-cm linear analogue scale) and (iii) presence of ulcerated or non-ulcerated stenosis were weighed and summed to yield a total score that ranges from 0 to 44.

Quantification of endoscopic lesion was calculated globally and per segment. Scoring a CDEIS per segment was performed to enable more accurate matching between MRE and endoscopy per segment. To determine a CDEIS per segment, the small bowel and the colon were divided into six segments: terminal ileum, right colon (cecum plus ascending colon), transverse colon, descending colon, sigmoid colon and rectum. Active disease was defined as CDEIS ≥ 3.

### CT Enterography Imaging Technique and image analysis

CT enterography imaging was performed as described in our previous report [10]. All patients were studied using a 64-detector CT scanner (GE Medical System, Milwaukee, WI, USA). Unenhanced and contrast-enhanced CT scans were performed in supine position from the diaphragm to the perineum during a single breath-hold. Contrast-enhanced CT imaging was performed by using 228 mAs, 120 kV, and 100 mL of intravenous contrast material (Lopamiro 370, Bracco Sine, Shanghai, China) at a rate of 3 mL/s. Scanning parameters were as follows: collimation 40 mm, pitch 1.375:1, and reconstruction thickness 1.5 mm. Enteric phase was conducted 70 s after administration of contrast agent, and delayed phase was conducted 20 s later.

The images evaluation and measurements were made on a workstation (GE Workstation 4.4). CTE findings and subjective image quality were reviewed by one experienced gastrointestinal radiologist independently who were blinded to all the clinical endoscopic, or biological information. CTE parameters evaluated in this study included mural hyperenhancement, mural thickening, ulceration, polyp, mural stratification, submucosal fat deposition, sacculation of antimesenteric wall, stricture, comb sign, perienteric fat hypertrophy and mesenteric fibrofatty proliferation.

To determine mural thickening, the inner and outer diameters of the bowel lumen in the loop most distended in each segment were measured. For calculating mural thickness, the inner diameter was subtracted from the outer diameter and divided by two [12]. All bowel wall attenuation measurements were made using a round 25-pixel region of interest (ROI) placed over the bowel wall. The bowel wall attenuation was determined by slowly moving the ROI around the circumference of the wall, keeping the ROI over the wall. The highest attenuation measured was the absolute attenuation used for the analysis [12]. Mural stratification includes bilaminar or trilaminar appearance to the bowel wall. Bilaminar mural stratification refers to mucosal hyperenhancement and decreased intramural attenuation. Trilaminar mural stratification refers to alternating areas of high and low attenuation due to mucosal and serosal hyperenhancement and low intramural attenuation. The comb sign is defined by CT features of segmental dilatation of the vasa recta involving a bowel loop. Perienteric fat infiltration is defined as focally and increased inhomogeneous attenuation in the perienteric fat, compared with the perienteric fat adjacent to non-inflamed bowel loops. Fibrofatty proliferation refers to fatty deposition along the mesenteric border of bowel segments affected by CD. Mesenteric lymph nodes located near the affected intestinal segments are considered pathological if their transverse diameter is > 10 mm. Image noise was determined by the average of five 100 mm^2^ ROIs placed on background. Moreover, relative attenuation was calculated by absolute difference of attenuation between post-contrast loop attenuation and pre-contrast loop attenuation, then divided by pre-contrast loop attenuation and adjusted by relative noise ratio. According the following formula: relative attenuation = ((postcontrast loop attenuation − precontrast loop attenuation)/(precontrast loop attenuation)) × 100 × (noise precontrast/noise postcontrast).

### Statistical analysis

All statistical analyses were conducted using the SPSS V.20 and the STATA V.12.0. A p value of less than 0.05 (two tailed) was considered statistically significant.

Quantitative variables included mural hyperenhancement and mural thickness. Differences in quantitative measures were tested by variance (ANOVA) followed by the Bonferroni post-hoc test, and differences in qualitative variables were compared by exact Fisher's test or χ^2^ test. If data was not normal distribution, Mann Whitney U test was explored. For the purpose of defining independent predictors of disease activity, a binary logistic regression was carried out with the presence of an endoscopic lesion (of any type) or ulcers at endoscopy or CDEIS ≥ 3 as the dependent variable, and mural thickening, mural stratification, perienteric fat infiltration and comb sign at CTE as independent variables. Considering the values for CEDEIS were non-normal distributed censored data, a Tobit regression model was employed with the calculated CDEIS per segment as the dependent variable, and the same CTE variables listed above as independent variables. The accuracy of the scores determined by CTE was assessed by calculating receiver operating characteristic (ROC) curves.

Correlations between the CDEIS, Harvey–Bradshaw index, CRP and CT index score were measured by the Spearman rank coefficient.

### Statistical power

We estimated that by studying 45 patients at least 200 ileocolonic segments would be available for examination. This number provides the ability to detect differences of 75% for normal bowel segments and 25% for the presence of an endoscopic lesion (of any type) with an alpha risk of 0.05 and beta of 0.1.

## Results

### Demographic and clinical data

**Fig. 1:**
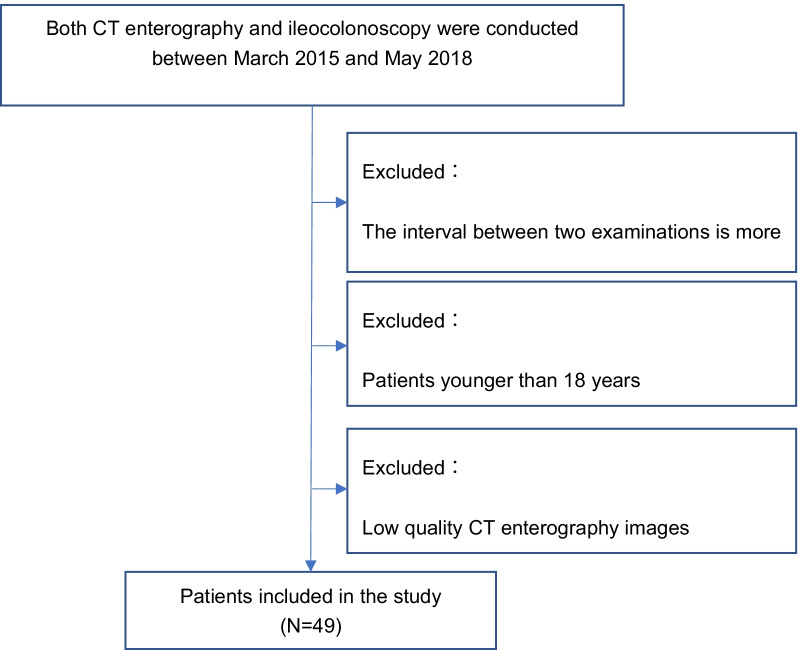
Flow chart of patient selection

Between March 2015 and May 2018, forty-nine patients were recruited, and a total of 280 intestinal segments were evaluated, including 49 rectum, 49 sigmoid colon, 49 descending colon, 48 transverse colon, 46 ascending colon and 39 distal ileum. A flow chart of patient selection is presented in Fig. 1. All patients underwent CTE and ileocolonoscopy. All included patients had good quality CTE images. The baseline demographic data were presented in Table 1. Thirty (61.2%) were men (median age, 26 years; age range, 18–43 years), and 19 (38.8%) were women (median age, 26 years; age range, 18–69 years). The median age at the inclusion is 27 (18–69) years old, and the median disease duration was 5 (1–145) months. The median C-reactive protein level was 18.2 (0.16–113) mg/l and was elevated relative to reference values (< 8 mg/l) in 33 patients. The median HBI was 5 (range 1–10). According to the Harvey–Bradshaw index, 23 patients were in clinical remission (index ≤ 4) and 26 patients had active disease (index > 4). According to Montreal's classification, 12.2% were classified as L1 (ileal), 14.3% as L2 (colonic) and 73.5% as L3 (ileocolonic).

**Table 1:** Baseline characteristics of the 49 Crohn’s disease patients

Male gender (*n*, %)	30 (61.2%)
Median age at the inclusion (years, range)	27 (18–69)
Median disease duration (months, range)	5 (1–145)
Previous intestinal resection (*n*, %)	3 (6.1%)
Disease location	
Ileal, n (%)	6 (12.2%)
Ileocolonic, n (%)	36 (73.5%)
Colonic, n (%)	7 (14.3%)
Perianal involvement, n (%)	32 (65.3%)
Concomitant therapies	
5-ASA (n, %)	31 (63.3%)
Steroids (n, %)	27 (55.1%)
Immunosuppressives (n, %)	31 (63.3%)
Anti-TNF antibodies, n (%)	27 (55.1%)
Median CRP (mg/l, range)	18.2 (0.16–113)
Harvey–Bradshaw index, median (IQR)	5 (1–10)
Harvey–Bradshaw index > 4, n (%)	26 (53.1%)
CDEIS, median (IQR)	9.5 (0.3–25.5)

Complete endoscopic evaluation of the colon with intubation of the ileum through the ileocecal valve was achieved in 39 patients (83%). Reasons for incomplete ileocolonoscopy were disease severity (n = 1) and stenosis (n = 9). Of these, 98 segments were normal, 61 segments had mild lesions including erythema, pseudopolyps or aphtoid ulcers, and 121 segments had severe lesions with superficial and/or deep ulcers.

### CT findings according to endoscopic lesion severity

According to the prevalence of qualitative findings for CTE values (Table 2), 93 segments exhibited ulcerations, 37 for polyp, 129 for mural stratification, 10 for submucosal fat deposition, 7 for sacculation of antimesenteric wall, 2 for stricture, 38 for comb sign, 65 for mesenteric fibrofatty proliferation, and 16 for perienteric fat hypertrophy. Mural stratification on CT examination were present in 81.8% of ileocolonic segments with ulcers at endoscopy and in 70.1% of CDEIS ≥ 3 at endoscopy, which were significantly less frequent in segments with inflammatory lesions without ulcers (39.3%) and in endoscopically normal segments (6.1%). Ulcerations on CT examination were identified in 64.5% of ileocolonic segments with ulcers at endoscopy and in 51.1% of CDEIS ≥ 3 at endoscopy, which were significantly less frequent in segments with inflammatory lesions without ulcers (19.7%) and in endoscopically normal segments (3.1%). Comb sign were identified by CT examination in 28.9% of patients with ulcers at endoscopy and in 21.3% of CDEIS ≥ 3 at endoscopy, which were very rare in segments with mild inflammation (3.3%) and in endoscopically normal mucosa (1.0%). The presence of submucosal fat deposition, sacculation of antimesenteric wall, perienteric fat hypertrophy, and polyp were less prevalent (Table 3).

**Table 2:** CTE findings for each ileocolonic segments in the 49 included Crohn’s disease patients

	Distal ileum n = 39	Ascending colon n = 46	Transverse colon n = 48	Descending colon n = 49	Sigmoid colon n = 49	Rectum n = 49
Ulceration	20	13	21	19	15	5
Polyp	0	11	12	12	2	0
Mural stratification	30	24	31	24	12	8
Submucosal fat deposition	3	2	1	1	1	2
Sacculation of antimesenteric wall	3	1	2	1	0	0
Strictures	1	0	1	0	0	0
Comb sign	6	3	8	10	9	2
Perienteric fat hypertrophy	7	4	5	0	0	0
Mesenteric fibrofatty proliferation	11	15	14	15	7	3
Mural hyperenhancement (HU)	194.13 + 122.23	127.51 + 70.69	153.38 + 101.19	125.16 + 93.15	108.5 + 94.3	98.4 + 86.2
Mural thickness (mm)	4.57 + 2.51	4.22 + 3.27	4.08 + 2.75	3.68 + 2.37	2.92 + 1.87	2.54 + 1.82

**Table 3:** Prevalence of qualitative CTE findings according to endoscopic severity of ileocolonic lesions

	Normal mucosa	Non-ulcerative lesions	Ulceration	Active disease at endoscopy (CDEIS ≥ 3)
No(N = 280)	98	61	121	174
Mural stratification	6 (6.1%)	24 (39.3%)	99 (81.8%)	122 (70.1%)
Ulceration	3 (3.1%)	12 (19.7%)	78 (64.5%)	89 (51.1%)
Polyp	2 (2.0%)	13 (21.3%)	22 (18.2%)	35 (20.1%)
Submucosal fat deposition	1 (1.0%)	3 (4.9%)	6 (5.0%)	9 (5.2%)
Sacculation of antimesenteric wall	0	3 (4.9%)	4 (3.3%)	7 (4.0%)
Perienteric fat hypertrophy	1 (1.0%)	3 (4.9%)	12 (9.9%)	15 (8.6%)
Perienteric fat stranding	2 (2.0%)	9 (14.8%)	54 (44.6%)	63 (36.2%)
Comb sign	1 (1.0%)	2 (3.3%)	35 (28.9%)	37 (21.3%)

**Fig. 2:**
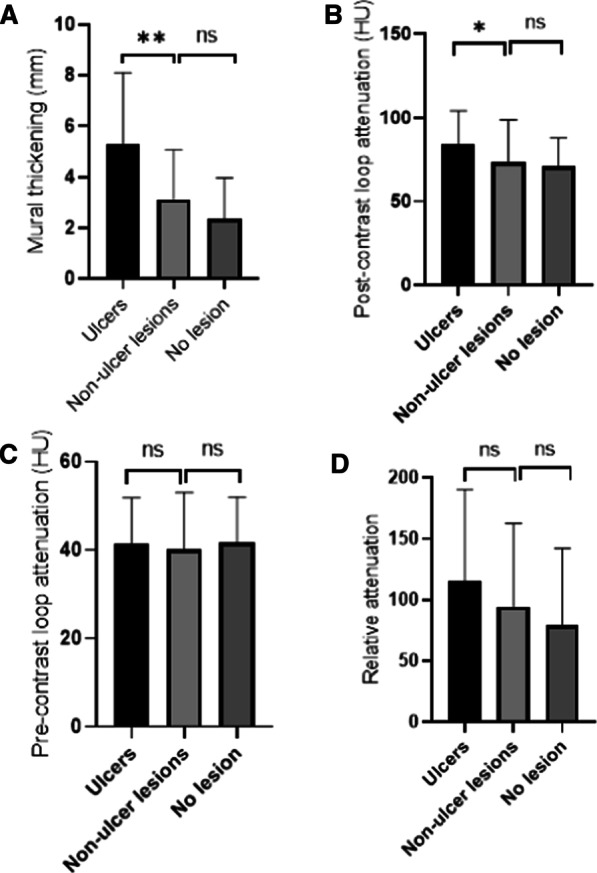
Changes in quantitative CTE parameters according to endoscopic severity of ileocolonic lesions. **A** mural thickness, **B** post-contrast loop attenuation, **C** pre-contrast loop attenuation, **D** relative attenuation. *p < 0.01, ** p < 0.001

Figure 2 depicted the changes in quantitative CT parameters according to endoscopic severity of ileocolonic lesions. Mean (SD) mural thickness was significantly higher in segments with ulcers (5.31[2.79] mm) compared in segments with inflammatory lesions (p < 0.001), but similar between segments with normal mucosa (2.37[1.59] mm) and segments with non-ulcer lesions (3.12[1.95] mm). In contrast, mean (SD) bowel wall attenuation was similar in all groups (p = 1.0) before intravenous injection of contrast, 41.52(10.31)HU for segments with ulcer, 40.17(12.87) HU for segments with non-ulcer lesions, and 41.82(10.18) HU for segments with normal mucosa. After intravenous injection of contrast, mean (SD) bowel wall attenuation was markedly elevated in segments with ulcers (84.16[20.01]HU) relative to segments with inflammation without ulcers (73.42[25.39]HU) (p = 0.004). Meanwhile, there was significant difference between segments with ulcers (115.45[74.72]) and segments with normal mucosa (79.20[62.92]) for relative attenuation (p < 0.001).

### Predictors of disease activity and severity in CTE

To determine which of the CT findings had an independent predictive value for the presence of an endoscopic lesion (of any type) or ulcers at endoscopy or CDEIS ≥ 3 for a particular segment, a binary logistic regression was used.

**Fig. 3:**
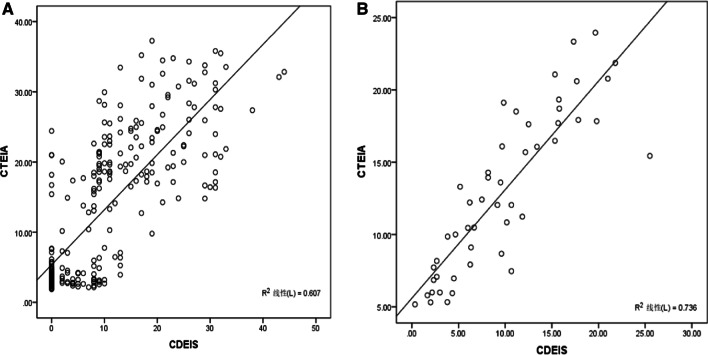
Correlation between the Crohn’s Disease Endoscopic Index of Severity (CDEIS) and CTEIA on a per-segment (**A**) and on a per-patient basis (**B**)

The CTE findings of predicting the presence of an endoscopic lesion (of any type) independently were mural thickness (p < 0.001) and mural stratification (p = 0.001). The accuracy of a score based on these variables to predict an endoscopic lesion was high, with an area under the ROC curve of 0.859, and sensitivity 0.78 and specificity 0.89 (Additional file 1: Fig. S1A). Logistic regression analysis showed that independent predictors of the presence of ulcers at endoscopy were mural thickness (p < 0.001) and mural stratification (p = 0.001). An area under the ROC curve is 0.905 for the score based on these variables to predict the presence of ulcerations at endoscopy, with sensitivity of 0.82 and specificity of 0.84 (Additional file 1: Fig. S1B). Mural thickness (p < 0.001) and mural stratification (p = 0.001) were independent predictors of the presence of an endoscopic lesion (CDEIS ≥ 3) with an area under the ROC curve of 0.859, and sensitivity 0.78 and specificity 0.89. Since data distribution for segmental CDEIS was not normal, with 35% of segments having no significant lesions, a Tobit regression analysis was applied. This analysis demonstrated that independent predictors for CDEIS were mural thickness (p < 0.001), mural stratification (p < 0.001) and comb sign (p = 0.002). From the analysis, we derived a simplified CT enterography index (CTEIA) to quantify disease activity based on CTE findings in each segment as follows: CTEIA (segment) = 2.1 mural thickness(mm) + 9.7 mural stratification + 5.2 comb sign. There was a high and significant correlation coefficient between the CDEIS of the segment and the CTE index calculated according to the logistic regression analysis coefficients (r = 0.779, p < 0.001) (Fig. 3A).

**Fig. 4:**
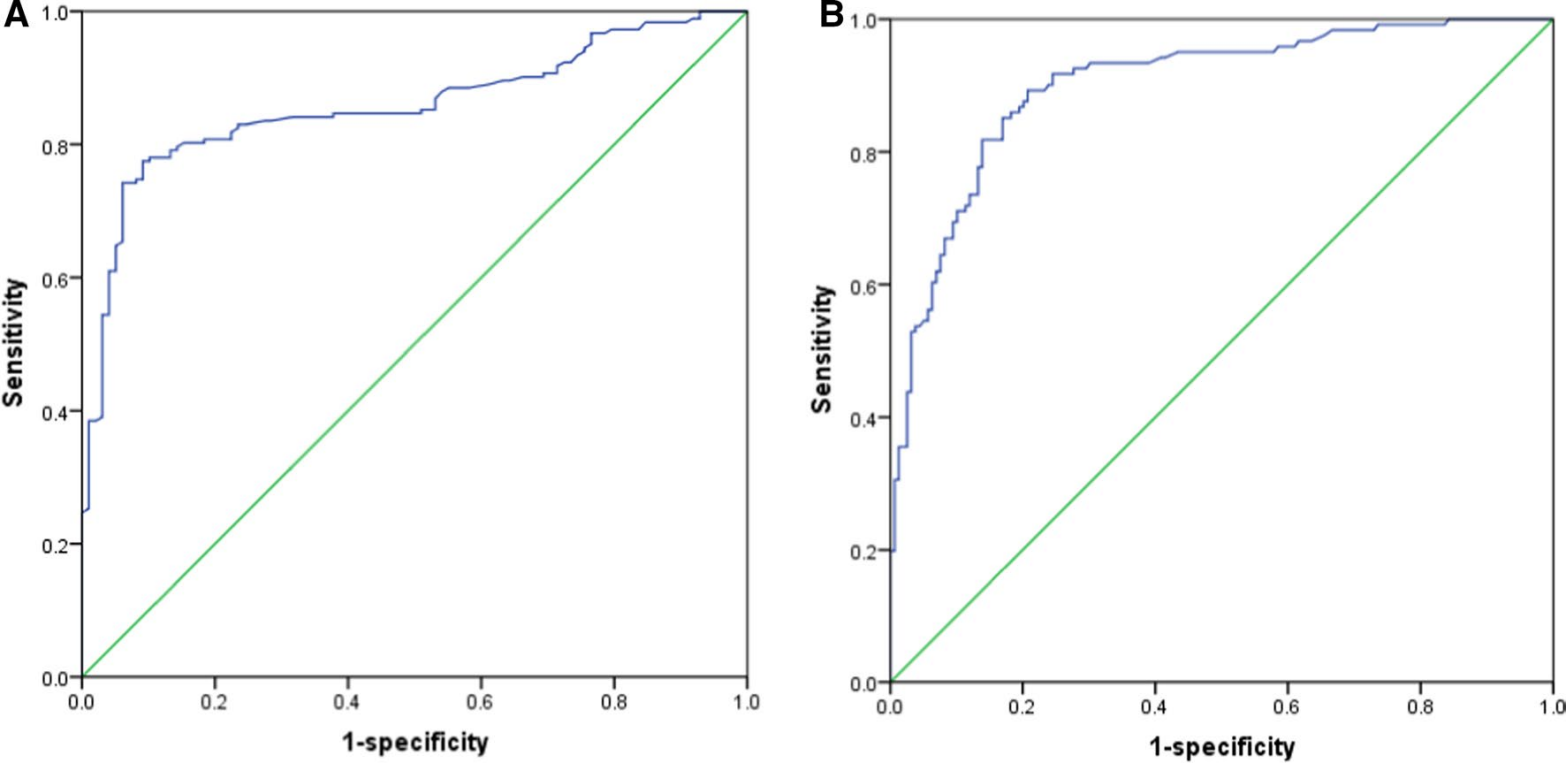
Receiver operating characteristic (ROC) curves of a CTEIA to predict presence of an endoscopic lesion (**A**) and presence of ulcerative lesions (**B**) in Crohn’s disease

**Fig. 5:**
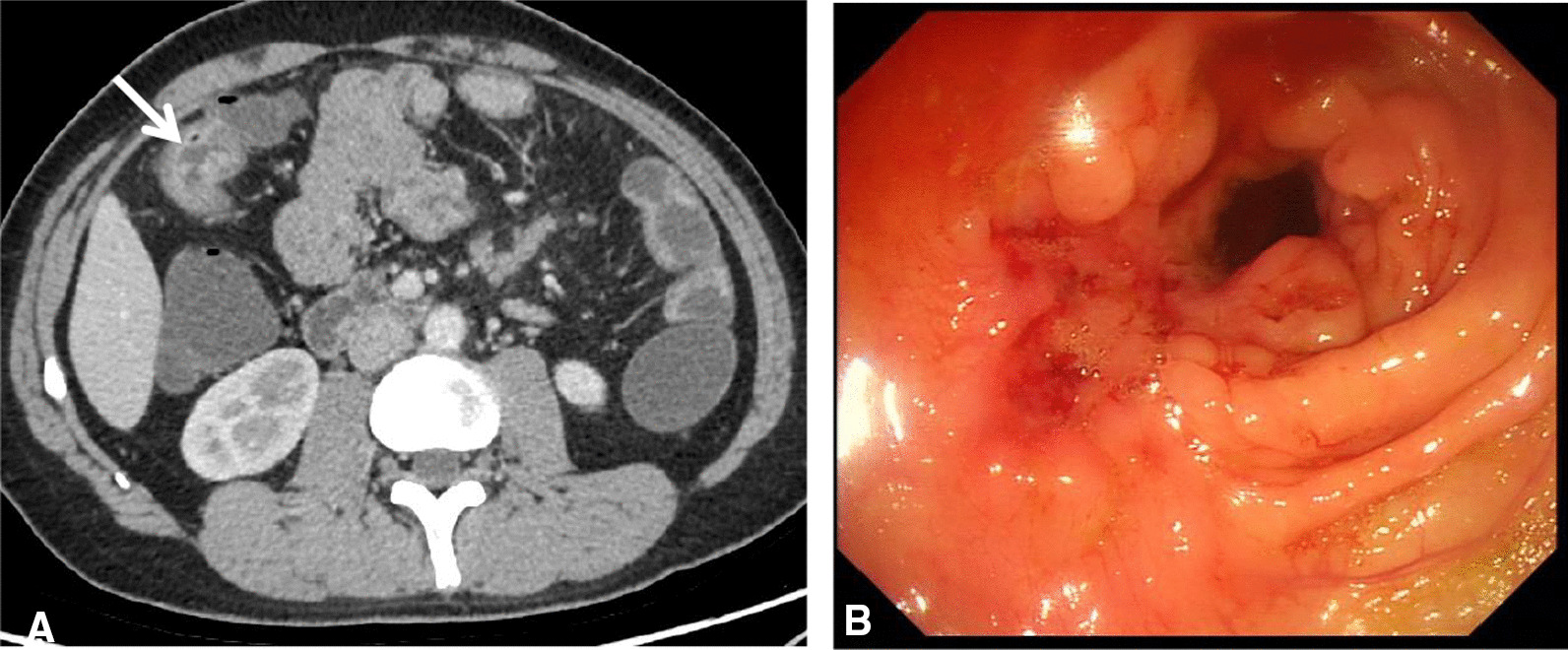
A 30-year-old woman with severe Crohn’s disease, **A** axial multidetector CT image of the transverse colon shows abnormal wall thickening, transmural enhancement (full arrow) and ulcer (white arrow) with CTEIA score 18; **B** view at ileocolonoscopy shows longitudinal ulcer in the same segment with CDEIS score 29

CTEIA score range was 1.88–37.26. The index has a high accuracy for the detection of an endoscopic lesion (area under the ROC curve 0.857, sensitivity 0.775, specificity 0.908, using a cut-off point of 6.25) (Fig. 4A) and for the detection of ulcerative lesion (area under the ROC curve 0.901, sensitivity 0.851, specificity 0.83, using a cutoff point of 13.96) in the colon and terminal ileum (Fig. 4B). Examples of CTE alterations associated with the presence of active inflammation were shown in Fig. 5.

### Comparison of CTE and other measures of disease activity

Correlations between the CDEIS, Harvey–Bradshaw index, CRP and CTEIA were measured by the Spearman rank coefficient. A global CTEIA was calculated by adding the values of rectum, sigmoid, descending, transverse and ascending colon and ileum. A significant correlation of the global CTEIA was observed with CDEIS (r = 0.858, p < 0.001) (Fig. 3B), Harvey–Bradshaw index (r = 0.455, p = 0.001) and CRP (r = 0.634, p < 0.001). Correlations of CDEIS with the Harvey–Bradshaw index (r = 0.452, p = 0.001) and CRP (r = 0.763, p = 0.001) were of similar magnitude to those of the CTEIA.

## Discussion

Mucosal healing is considered as the main treatment goal for Crohn's disease which is limited to the assessment of the mucosa. However, CD is a transmural inflammatory process which also involves the mesentery. Therefore, wall thickening, muscular hypertrophy and mesenteric hypertrophy with fat accumulation and hypervascularization are characteristic features of CD [13]. Many studies have focused on developing cross-sectional imaging indices for quantification of active CD. These indices were developed to standardize measured outcomes in clinical trials of treatment interventions for CD. Rimola et al. [3] were the first to develop MaRIA (Magnetic Resonance Index of Activity), included following parameters: bowel wall thickness, ulcers, edema, relative contrast enhancement of the intestinal wall. Neye et al. [14] developed an US index with bowel wall thickness and Doppler signal as parameters. In Europe, there is increased emphasis on MR enteography and ultrasound over CT enterography for imaging Crohn’s disease [15]. However, MR enterography is limited by cost, long acquisition times, a lack of availability and the expertise necessary to properly interpret the images [16]; US has some limitations, such as operator dependence, bowel gas, obesity and difficulty in exploring the entire intestine [17].

In this study, mural thickness and mural stratification were independent predictors of the presence of an endoscopic lesion (of any type) or ulcers at endoscopy or CDEIS ≥ 3. As previous studies reported, mural hyperenhancement, mural thickness, mural stratification, segmental mural hyperenhancement, increased density of mesenteric fat, and comb sign have been shown to correlate with active inflammation. Colombel et al. [18] showed that endoscopic score was significantly correlated with CT bowel enhancement, comb sign, and fat density (Spearman correlation coefficients 0.33–0.39; p < 0.001). Bodily et al. [19] also showed that mural hyperenhancement had the highest sensitivity for predicting the presence of active inflammatory disease, with a sensitivity of 80% for patients with definite active Crohn disease. Sakurai et al. [20] demonstrated that CTE findings obtained from the mesenteric area, such as comb sign and enlarged mesenteric lymph nodes, were more critical predictors of endoscopic mucosal ulceration than those obtained from the bowel wall.

In addition to prediction, CTE appeared to be a useful diagnostic method for assessment of mucosal healing and treatment response in Crohn’s disease. Hashimoto et al. [21] showed that mucosal findings showed an association with ulcer in 93.6% of active group patients but in only 12.5% of inactive group patients (p < 0.0001), whereas mucosal healing was found in 62.5% of inactive group patients but in only 3.2% of active group patients (p < 0.0001). Wu et al. [22] determined whether CTE changes of Crohn’s disease after treatment correlated with clinical remission. After treatment, bowel wall thickening was attenuated in 88% of CD patients. Thickness of bowel wall was decreased from 8.8 ± 2.8 mm to 6.4 ± 1.9 mm (P < 0.001). CT value of bowel wall in portal stage was also declined from 90.0 ± 15.4 (HU) to73.4 ± 14.2 (HU) (P < 0.001).

As MaRIA index from MRE, this is the first time to establish a new scoring system to evaluate disease activity in patients with ileocolonic Crohn's disease. In the formula, three parameters (mural thickness, mural stratification and comb sign) were included. For segment analysis, the index had a high accuracy for the detection of an endoscopic lesion (area under the ROC curve 0.857, sensitivity 0.775, specificity 0.908, using a cut-off point of 6.25) and for the detection of ulcerative lesions (area under the ROC curve 0.901, sensitivity 0.851, specificity 0.83, using a cutoff point of 13.96) in the colon and terminal ileum. For per-patient analysis, a significant correlation of the global CTEIA was observed with CDEIS (r = 0.858, p < 0.001), Harvey–Bradshaw index (r = 0.455, p = 0.001) and CRP (r = 0.634, p = 0.000).

The new quantitative CTE index of disease activity and severity in CD might be applied in clinical practice and research studies, for example, correlated with patient prognosis, mutual conversion with MRE score, etc. Shyn et al. [23] low-dose (18)F-FDG PET/CTE, compared with CTE, might improve the detection and grading of active inflammation in patients with Crohn disease. CTEIA could also be used as a supplement to pet score.

As we all know, the most obvious disadvantage of CT is ionizing radiation. Therefore, utilization of CTE for the imaging of bowel diseases for several reasons: 1) Newly diagnosed patients; 2) CTE is of course undoubtedly used particularly in older patients; 3) CT is ubiquitous and widely available and accessible in most emergency rooms and outpatient radiology practices. It is worth mentioning that low-dose CT techniques such as iterative reconstruction and kV selection are now widely available [15].

Some limitations in our study should be acknowledged. Firstly, this is a retrospective study performed at a single center, with a limited number of endoscopists and radiologists evaluating the diagnostic tests. Interobserver variability did not assessed in the study. Secondly, data about radiation exposure was not collected. Thirdly, our medical history system only records HBI score and lacks CDAI data. Finally, there is lack of anisodamine injection before CTE owing to our institute policy.

## Conclusions

The current study provides a new CTE index of disease activity and severity in CD, and it also support the implementation of the quantitative index in research studies. Prospective validation, predicting mucosal healing, low-dose CT enterography could be explored in future studies.

## Supplementary Information

**Additional file 1. Fig. S1**: Receiver operating characteristic (ROC) curves of a CT-based score to predict presence of an endoscopic lesion (A) and presence of ulcerative lesions (B) in Crohn’s disease.

## Data Availability

The data of this study is available from the corresponding author.

## Abbreviations

CTE: CT enterography
CD: Crohn's disease
CDEIS: Crohn's disease endoscopic index of severity
SES-CD: Simple endoscopic score for Crohn's disease
ROC: Receiver operating characteristic
CTEIA: CT enterography index (CTEIA)
