# Supplementary material for: CT enterography for evaluation of disease activity in patients with ileocolonic Crohn's disease
Source: BMC Gastroenterol. 2022 Jun 30;22:324. doi: 10.1186/s12876-022-02389-5 (PMC9248101; doi:10.1186/s12876-022-02389-5)
Supplement: Supplementary file 1 — Additional file 1. Fig. S1: Receiver operating characteristic (ROC) curves of a CT-based score to predict presence of an endoscopic lesion (A) and presence of ulcerative lesions (B) in Crohn’s disease. [file 12876_2022_2389_MOESM1_ESM.docx]

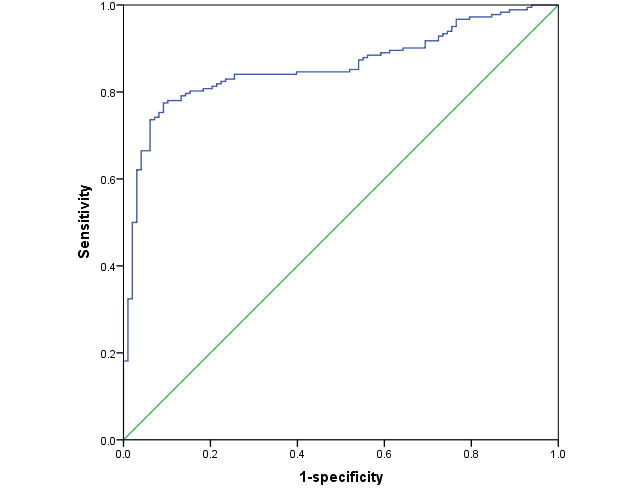

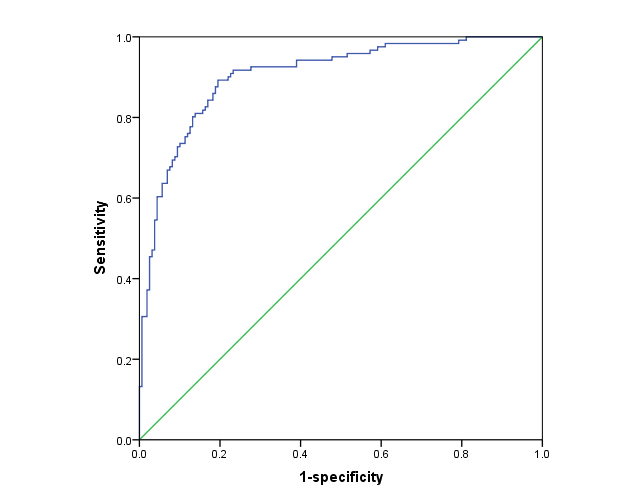


**A**

**B**

**Fig. S1**

Receiver operating characteristic (ROC) curves of a CT-based score to predict presence of an endoscopic lesion (A) and presence of ulcerative lesions (B) in Crohn’s disease.
